# Assessing Awareness of Genetic Risks among Pregnant Mothers in Consanguineous Marriages: A Cross-Sectional Study in Government Healthcare Settings

**DOI:** 10.34763/jmotherandchild.20263001.d-26-00013

**Published:** 2026-05-31

**Authors:** Saim Mahmood Khan, Jawairiya Muhammad Hussain, Laiba Bukhari, Mahnoor Khan, Zarnab Saleem, Aroobah Jawwad, Mahnoor Sheikh, Fizzah Iftikhar, Surraiya Riaz Mahmood Khan

**Affiliations:** Karachi Medical and Dental College, MBBS, Karachi, Sindh, Pakistan; Khyber Girls' Medical College, MBBS, Peshawar, KP, Pakistan; CMH Lahore Medical College and Institute of Dentistry, MBBS, Lahore, Punjab, Pakistan; Services Institute of Medical Sciences, MBBS, Lahore, Punjab, Pakistan; Jinnah Sindh Medical University, MBBS, Karachi, Sindh, Pakistan; Sohail University, Pharm D, Karachi, Sindh, Pakistan

**Keywords:** Marriage, risks, pregnancy, genetic awareness, cousin marriages, congenital anomalies

## Abstract

**Background:**

Consanguineous-marriages are highly prevalent among the Pakistani population, leading to an increased risk of genetic disorders in offspring. However, awareness of these genetic risks remains low, which delays diagnosis and contributes to poor health outcomes. This study's objective is to assess awareness of the genetic risks of consanguineous marriage and examine its effects on pregnancy outcomes.

**Materials and methods:**

A descriptive cross-sectional-study was conducted among 365 pregnant women attending antenatal clinics in Peshawar. Data were collected using a self-structured questionnaire. Sample size was calculated using an estimated prevalence of 36% from prior NCBI-indexed studies. Participants were selected through systematic random sampling, including women in consanguineous marriages who consented to participate. Data were analyzed using descriptive statistics and Chi-square test, with p ≤ 0.05 considered statistically significant.

**Results:**

Among 365 pregnant women, 41.0% had low awareness of genetic risks, 29.3% had no knowledge, and only 10.6% demonstrated good understanding of consanguineous marriage. Genetic screening was reported by 20.4%. Higher awareness was associated with older age, higher education, and prior screening. Family history of genetic disorders was significantly associated with age (p = 0.003), education (p = 0.010), duration of marriage (p = 0.003), awareness (p = 0.004), prior screening (p < 0.001), and consanguinity (p = 0.011). It was strongly linked to adverse pregnancy outcomes, including miscarriages, stillbirth, and developmental delay (p < 0.001).

**Conclusion:**

Awareness of genetic risks in consanguineous marriages is low among pregnant women in this setting. Improving education and genetic counseling during antenatal care could help increase understanding and screening.

## Introduction

Consanguineous marriage — i.e., between individuals who are closely related genetically — has been a longstanding practice across human history. It is highly prevalent in some cultures and communities, and raises significant concerns regarding the increased risk of genetic disorders in children. Consanguineous marriages are common in developing countries, but their frequency has declined in developed countries. Globally, it is estimated that more than 10% of the population is involved in consanguineous unions, with particularly high prevalence in Asia, North Africa, and the Middle East [1]. However, the Southeast Asia region still shows a high rate of inter-cousin marriages. Studies from various countries and communities in Southeast Asia show the prevalence of cousin marriages ranging from 20% to 70%. They are often maintained for sociocultural and economic reasons, such as strengthening family ties, maintaining social cohesion, and facilitating intergenerational wealth preservation.

In clinical genetics, consanguineous marriage refers to a union between individuals who are closely related, with an inbreeding coefficient (F) of 0.0156 or greater. This coefficient reflects the proportion of genetic loci at which offspring are likely to inherit identical alleles from both parents due to shared ancestry [2]. In populations where consanguineous unions are common, partners are significantly more likely to carry the same recessive genetic variants, increasing the incidence of autosomal recessive disorders in their offspring [3]. Studies from the South Asia region show prevalence rates of consanguinity ranging from 20% to over 60% [4]. Such genetic overlap elevates the likelihood of homozygosity for deleterious recessive alleles, thereby increasing the risk of inherited genetic disorders, including autosomal recessive syndromes, congenital anomalies, and intellectual disabilities [5]. Given these implications, the intersection of cultural practice and biomedical risk necessitates a closer examination of public awareness, particularly among women during critical reproductive periods, including pregnancy.

Pakistan is a country where consanguineous marriages are a societal norm and are widely practiced. Previous studies have shown that in Pakistan, consanguinity is present in approximately 60% of marriages, of which 80% are between first cousins [6]. Inherited disorders have been found to be twice as common in consanguineous marriages as they are in non-consanguineous marriages [7]. A study conducted in Karachi in a public hospital setting showed that consanguinity among parents was present in 72.7% of the admitted patients in the pediatrics ward. Among them, 87% of parents were first cousins, while 12% were second cousins [8]. Additionally, a community-based study from India demonstrated a 36% prevalence of consanguinity, and this figure was adopted for our sample size calculation in the present study [9].

Despite the well-known correlation between consanguinity and genetic disorders, there exists a knowledge gap in studies focusing on awareness levels, particularly within the Pakistani community, where consanguineous marriages are highly prevalent. Public-sector hospitals serve a large portion of the population, particularly those from socioeconomically disadvantaged backgrounds, who are more likely to engage in consanguineous marriage. Information regarding the use of genetic counseling and screening services during prenatal care is also limited in the existing literature. In light of this, this study aims to evaluate awareness of genetic risks associated with consanguineous marriage among pregnant women attending government hospitals to guide targeted antenatal education and evidence-based public health policies.

It is important to recognize that consanguineous marriages are closely linked to cultural, social, and economic constructs. Therefore, rather than trying to challenge such cultural norms, it is essential to focus on increasing awareness, supporting informed reproductive decisions, and improving access to genetic counseling and screening services.

## Materials and methods

### Study design and setting

This study was carried out as a cross-sectional hospital-based study at the prenatal outpatient clinics of Hayatabad Medical Complex in Peshawar, Pakistan (HMC). Its objective was to evaluate the level of genetic risk awareness among pregnant women in consanguineous marriages.

### Study duration

Data collection was carried out over a period of four months, from August 2025 to November 2025. Antenatal outpatient clinics were chosen as the study setting because they provide access to a large number of pregnant women from different socioeconomic and educational backgrounds.

### Participants

The participants were pregnant women in consanguineous marriages who visited antenatal clinics during the study period. In this study, consanguineous marriage was defined as a marital union between two individuals who are second cousins or closer relatives.

### Sample size

The sample size was calculated using the Raosoft sample size calculator [10] with a confidence level of 95%, a margin of error of 5%, and an expected awareness prevalence of 36%, based on previously published regional studies [9]. The minimum calculated sample size was 348 participants. To improve reliability and to compensate for incomplete responses, a total of 365 participants were included in the final analysis.

### Sampling technique

A systematic random sampling method was used to select participants. Every third pregnant woman attending the antenatal clinic who was eligible for the study according to inclusion criteria was invited to participate.

### Inclusion criteria

Currently pregnant women

- In consanguineous marriages (up to fifth-degree relatives)
- Attending the antenatal clinics at HMC Peshawar during the study period
- Willing to participate and able to provide informed consent.

### Exclusion criteria

- Women in non-consanguineous marriages
- Women who declined to participate
- Women who were severely ill or unable to complete the interview

### Data collection

Data were collected using a structured questionnaire, and interviews were carried out by research assistants. The questionnaire was developed after reviewing relevant literature.

In order to gather information around the subject of consanguineous marriages, the questionnaire was divided into various sections. These sections include sociodemographic factors, marital history, obstetric history, knowledge and awareness regarding genetic factors, attitudes and opinions regarding consanguineous marriages, and experiences with the healthcare system.

The questionnaire was validated by subject experts from the relevant field to ensure the validity of the content, and was pre-tested with 20 pregnant women. Pregnant women attending prenatal clinics of a private hospital were selected as the sample population. Interviews with participants were conducted in English, Urdu, or a local language, depending on the participant's preference.

### Study outcomes

The primary outcome of this study was to assess the level of awareness of genetic risks among pregnant women in consanguineous marriages.

Participants' level of awareness was determined through knowledge-based questions included in Sections C and D of the questionnaire. For each correct response, one point was given, while incorrect or “not sure” responses received zero points. A total score was determined for each participant based on their responses to these questions related to awareness.

The participants who correctly answered more than 50% of the questions related to awareness were placed in the “adequate awareness” category, whereas participants who correctly answered 50% or less of these questions were categorized as having “inadequate awareness.”

Besides assessing the level of awareness, other data related to socio-demographic and obstetric factors were also obtained through the questionnaire and assessed for their association with awareness levels of genetic risks.

### Data analysis

All collected data were entered and analyzed using the Statistical Package for Social Sciences (SPSS) version 25 [11].

Descriptive analysis was used for the study population. The descriptive analysis for continuous data, such as age, was performed by calculating the mean and standard deviation. The descriptive analysis for categorical data – such as education level, residential area, duration of marriage, family history of genetic disorders, and level of awareness – was performed by calculating the frequencies and percentages of each response.

Inferential analysis was conducted to determine the association between awareness of genetic risks and selected sociodemographic variables. A Chi-square test was used to determine the association between awareness level and certain selected variables, such as age group, education level, and family history of genetic disorders. A p-value ≤ 0.05 was considered statistically significant.

### Ethical considerations

Ethical approval was obtained from the Institutional Ethics Committee of Hayatabad Medical Complex, Peshawar, Pakistan (Approval No.: HMC/IRB/2025/2852) before data collection. Informed consent was given by each participant in the study. The study was entirely voluntary, and participants were allowed to leave at any time without facing any consequences. Anonymity and confidentiality of participant data were strictly maintained.

## Results

This study was conducted among 365 pregnant women who were all attending government hospitals for antenatal care. The sociodemographic profile of these women reflects their demographic characteristics and levels of awareness, as well as the critical link between family history and health outcomes.

### Sociodemographic profile of the participants

A total of 365 participants were recruited for the research. The majority of participants were above the age of 25 years (45.9%), followed by those between 22 and 25 years (30.4%). Those aged 18 to 21 years constituted 16.6%, while only 6.2% of the participants were below the age of 18. Regarding the education level of the participants, 30.4% of participants were educated at the college level or higher, while 21.5% had no education. Primary and secondary school-level education accounted for 16.6% and 17.4% of the participants, respectively, while 13.3% had a middle school-level education.

Almost half of the participants (48.1%) had been married for fewer than five years. Approximately 28.5% had marriage durations between five and 10 years. About 22.0% had been married for more than 10 years. When assessing participants' awareness level of genetic knowledge, 41.0% of them said they had “a little” knowledge about genetic risks, and 29.3% reported no knowledge at all. Only 10.6% said they understood what constitutes genetic risk “very well.” Genetic screening in a previous pregnancy was reported by only 20.4% of participants, while 78.8% had not undergone any screening. Consanguineous marriages among relatives were highly prevalent, having been reported by 85.1% of participants. The sociodemographic characteristics and awareness levels of the study participants are presented in Table 1.

**Table 1.** Frequency Table of Participant Characteristics.

**Characteristic**	**Frequency**	**Percentage**
**Age of participant**		
Less than 18 years	23	6.2
18–21 years	61	16.6
22–25 years	112	30.4
Above 25 years	169	45.9
**Level of education**		
No formal education	79	21.5
Primary school	61	16.6
Middle school	49	13.3
Secondary school	64	17.4
Higher education	112	30.4
**Years of Marriage**		
Less than 5 years	177	48.1
5 to 10 years	105	28.5
More than 10 years	81	22
**Understanding of the term “genetic risk”**		
A little	151	41
Fairly well	67	18.2
Very well	39	10.6
Not at all	108	29.3
**Genetic screening in previous pregnancy**		
Yes	75	20.4
No	290	78.8
**Consanguineous marriage in relatives**		
Yes	313	85.1
No	52	14.1

### Awareness and screening practices

A noticeable knowledge gap existed in participants' understanding of genetic risks and screening. A relatively small number of participants reported that they understand genetic risks “fairly well” (18.2%), and an even lower number reported that they understand the term “very well” (10.6%). In addition, approximately 70.3% of the participants indicated that they have “a little” or “no understanding” of the term. Participants had a low overall awareness of genetic risks. Most women either had limited knowledge or no knowledge at all, which may be reflected in the uptake rate of genetic screening and the reported frequency of genetic complications in pregnancies.

Bar chart showing the distribution of participant's self-reported understanding levels.

The majority of participants did not undergo genetic screening in their previous pregnancies. Only a small proportion (20.4%) reported having had genetic screening done, while a much larger group (78.8%) reported having had no genetic screening at all. This indicates that the rate of genetic screening practices is very low in the study population. Despite the high prevalence of consanguineous marriages and genetic risks, most women are not utilizing available screening services, and this may be linked to higher rates of obstetric and neonatal complications.

Bar chart illustrating the proportion of participants who underwent genetic screening versus those who did not.

### Association of sociodemographic variables with history of genetic disorders

There was a statistically significant association between a participant's history of genetic disorders and sociodemographic factors. The age of participants was significantly associated with genetic disorders (p = 0.003). Higher rates of genetic disorders were reported by participants above 25 years of age. Educational level also showed a significant association with a participant's history of genetic disorders (p = 0.010). Participants with higher education reported better awareness and demonstrated differing distributions in their reporting of genetic disorders. Years of marriage were also significantly associated with a history of genetic disorder (p = 0.003); it was observed that the longer the duration of marriage, the higher the occurrence of genetic disorders.

Understanding of the term “genetic risk” was also significantly related to a participant's history of genetic disorders (p = 0.004), and previous genetic screening also showed a highly significant association with a history of genetic disorders (p < 0.001). Participants who had a better understanding of genetic risk and had undergone screening were more likely to report a history of genetic disorders. Consanguineous marriage among relatives was significantly associated with a history of genetic disorders (p = 0.011). The associations between participants' sociodemographic variables and history of genetic disorders are summarized in Table 2.

**Figure 1.**
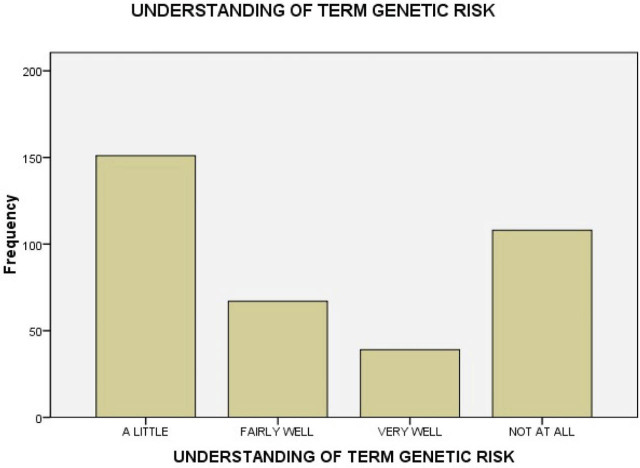
Understanding of the term “genetics risk” among participants.

**Figure 2.**
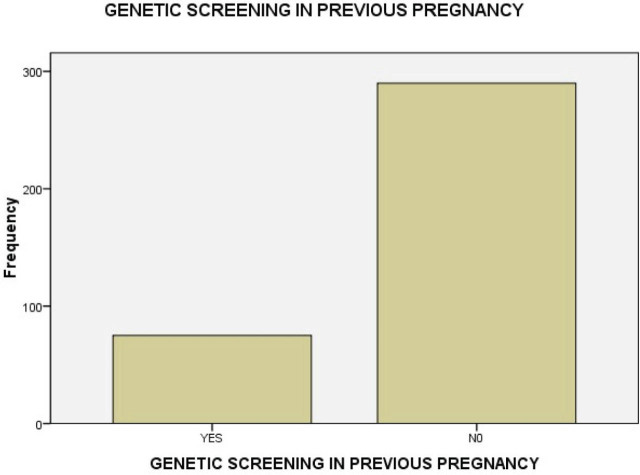
Genetic screening practices in previous pregnancies.

**Table 2.** Association of Sociodemographic Variables with History of Genetic Disorder.

**History Of Genetic Disorder**				
**Category**	**Yes**	**No**	**Not sure**	**P-value**
**Age of Participants**				
Less than 18 years	6	6	11	0.003
18-21 years	11	26	24	
22-25 years	14	70	28	
Above 25 years	37	96	36	
**Level of education**				
No formal education	18	34	27	0.010
Primary school	10	25	26	
Middle school	8	31	10	
Secondary school	8	41	15	
Higher education	24	67	21	
**Years of marriage**				
Less than 5 years	28	84	65	0.003
5 to 10 years	23	61	21	
More than 10 years	17	51	13	
**Understanding of term “genetic risk”**				
A little	27	74	50	0.004
Fairly well	19	35	13	
Very well	10	26	3	
Not at all	12	63	33	
**Genetic screening in previous pregnancy**				
Yes	28	35	12	0.000
No	40	163	87	
**Consanguineous marriage in relatives**				
Yes	66	163	84	0.011
No	2	35	15	

### Association between family history of genetic disorders and obstetric and neonatal complications

The statistical analysis revealed a significant association between family history of genetic disorders and obstetric and neonatal complications, with a probability of less than 0.001. The frequencies of miscarriage, stillbirth, congenital malformations, and developmental delays were higher in cases with family histories of genetic disorders compared to those without. Those without any complications were mostly participants that had no family history of genetic disorders. The relationship between family history of genetic disorders and obstetric and neonatal complications is shown in Table 3.

**Table 3.** Association between history of genetic disorders and obstetric/neonatal complications.

**Obstetric and neonatal complications**	**Family history of genetic disorders (number of participants)**			

	**Yes**	**No**	**Not sure**	**P-value**
Miscarriage	23	44	41	0.000
Stillbirth	5	12	7	
Congenital malformations	6	10	1	
Developmental delays	7	5	3	
No complications	27	127	47	

## Discussion

Our study found that pregnant women from consanguineous marriages who visited the prenatal clinics at Peshawar's Hayatabad Medical Complex knew very little about their genetic risk. The majority of participants demonstrated limited understanding of genetic risk, with very few even reporting familiarity with the concept. Additionally, uptake of genetic screening services was low, and consanguinity was highly prevalent in the study population. The most prominent barrier identified in our study was a lack of awareness of genetic risks, which likely limited the utilization of genetic screening services, followed by overall low uptake of such services.

One of the main conclusions of the study was that participants' understanding of genetic risks was insufficient. Only a small percentage of women said they understood the term “genetic risk.” Improving knowledge of genetic risks is therefore an important component of maternal health education.

Awareness appeared to be influenced by participants' education levels. Reporting of genetic disorders was significantly associated with educational attainment; women with higher levels of education were more likely to disclose a family history of genetic diseases and to express awareness of these conditions [8]. Women with higher levels of education were also more likely to participate in prenatal screening programs and were generally more aware of genetic conditions. History of genetic abnormalities was significantly associated with maternal age, exhibiting increased prevalence in individuals over 25. Other studies have found advanced maternal age to be associated with an increased risk of chromosomal abnormalities and adverse pregnancy outcomes [7]. In this study, a history of genetic disorders was strongly associated with consanguineous marriage; autosomal recessive disorders, such as hemoglobinopathies, metabolic diseases, and congenital anomalies, may be more common in populations with a high rate of such unions [4,5,11].

Consanguineous marriages were highly prevalent among the study participants, with more than 80% of women reporting having married cousins. Due to cultural traditions and social factors, consanguineous marriage is still common in many parts of the world. Any public health strategy must respect cultural values while promoting informed decision-making and risk awareness [12]. Similar trends have been observed in other low- and middle-income countries, where screening uptake is hampered by a lack of awareness, similarly to our study. These observations are consistent with previous research, which has found low awareness of genetic risks and limited use of prenatal screening services in similar populations [1,2,9,13]. Previous research has also identified other barriers, such as financial constraints and inadequate prenatal counseling. Structural factors, such as limited availability of testing facilities and trained genetic counselors, have also been found to contribute to low utilization of genetic screening services [14].

Effective prenatal counseling helps explain screening methods, genetic inheritance patterns, and pregnancy risks, supporting informed decision-making among couples [15]. Integrating genetic counseling into routine prenatal care, along with premarital and preconception programs in regions with widespread consanguinity, may further inform couples' decision-making. In regions with widespread consanguinity, premarital and preconception screening programs may reduce the incidence of inherited disorders – for example, hemoglobinopathies [16,5]. These findings are also supported by recent reviews highlighting the association of consanguinity with various genetic disorders [11].

Previous research has found that consanguinity increases the risk of inherited genetic diseases and birth defects. Communities with high levels of consanguinity have a higher prevalence of inherited hemoglobinopathies, and the incidence of miscarriage, stillbirth, congenital malformations, and developmental delays is higher among women with a positive family history of consanguinity than among those without. This is consistent with earlier studies linking genetic predisposition to adverse maternal and neonatal outcomes [4,17,18]. These findings highlight the importance of preventive strategies in reducing the transmission of inherited disorders, including premarital screening, preconception counseling, and integration of genetic counseling into routine maternal healthcare.

Communication between pregnant women and healthcare professionals can improve awareness and screening practices. Prenatal genetic counseling research focuses on clear communication and patient-centered approaches to improving understanding of genetic risks and screening options [15].

### Limitations

This study has several limitations that should be considered when interpreting its findings. First, the cross-sectional design makes it difficult to demonstrate causal links between genetic risk awareness and the occurrence of genetic disorders. Second, the study relied on self-reported data, which could be inaccurate because participants may not accurately recall the outcomes of previous pregnancies or their family history of genetic disorders. Third, the study was carried out in a single government tertiary-care facility, which restricts the findings' applicability to other healthcare settings. Furthermore, the relative contribution of each identified barrier to screening uptake was not quantitatively evaluated. Despite these limitations, the study provides valuable insights into genetic risk awareness among pregnant women in consanguineous marriages. When interpreting research on consanguineous marriage, however, cultural sensitivity and respect for individual autonomy must be taken into account.

### Future directions

Future research should further explore genetic awareness and its impact on maternal and neonatal health outcomes. Longitudinal studies may help clarify the relationship between awareness levels, consanguineous marriage, and pregnancy outcomes. Larger population-based studies may help identify high-risk groups and assist in the development of public health programs targeting those groups. Assessing community-based educational campaigns and integrating genetic counseling into routine antenatal care could help raise awareness and reduce the burden of hereditary genetic disorders [18,20,21,22].

## Conclusion

This research shows that there is a lack of awareness about the genetic risks for pregnant females in consanguineous unions, which are relatively common throughout the world. The absence of genetic screening puts women and their future babies at higher risk of developing congenital defects and complications during pregnancy. Maternal age and education were strongly correlated with awareness about genetic disorders, while previous genetic screenings were a determining factor in acquiring knowledge, showing that there are some missed opportunities in the healthcare system. From the results, it is clear that the problem is not just in lack of information, but in larger systemic failures to provide appropriate services and preventive healthcare policies. Targeted educational interventions, integration of genetic counseling into routine antenatal care, and policy-level efforts to improve access to affordable screening are urgently needed to reduce the burden of preventable genetic disorders.

### Key points

- Awareness of genetic risks among pregnant women in consanguineous marriages is dangerously low.
- Higher maternal age and education correlate with better awareness, but prior genetic screening is the strongest predictor.
- Systemic gaps in healthcare delivery, not only individual knowledge deficits, perpetuate the problem.
- Routine antenatal genetic counseling and affordable screening must become standard policy.
